# Therapeutic ultrasound in fracture healing: The mechanism of osteoinduction

**DOI:** 10.4103/0019-5413.42804

**Published:** 2008

**Authors:** P S John, C S Poulose, Benjamin George

**Affiliations:** Department of Orthopedics, Medical College, Kottayam, India; 1 Center for Neuroscience, CUSAT, India

**Keywords:** Fracture healing, neurotransmitter, thymidine incorporation, ultrasound

## Abstract

**Background:**

Ultrasound has been used therapeutically for accelerating fracture healing since many years. However, the controversy on the exact mechanism of osteoinduction still continues. In this study, we try to bring out the exact biomolecular mechanism by which ultrasound induces fracture healing.

**Materials and Methods:**

The study was conducted in two phases: animal experiments and clinical study. In the first phase, we induced fractures on the left tibia of Wistar strain rats under anaesthesia. They were divided into two groups. One of the groups was given low-intensity, pulsed ultrasound (30 MW/cm^2^) 20 min a day for 10 days. Tissue samples and radiographs were taken weekly for 3 weeks from both the groups. In the second phase of our study, ten patients with fractures of the distal end of the radius (ten fractures) were included. Five of these were treated as cases, and five were treated as controls. Ultrasound was given 30 MW/cm^2^ for 20 min every day for 2 weeks. The patients were assessed radiologically and sonologically before and after ultrasound therapy. Tissue samples were studied with thymidine incorporation test with and without adding various neurotransmitter combinations.

**Results:**

Radiological findings revealed that there was an increased callus formation in the ultrasound group. At the cellular level, there was an increased thymidine incorporation in the ultrasound group. When various neurotransmitters were added to the cells, there was an increased thymidine incorporation in the ultrasound group.

In the second phase of the study, radiological and sonological assessments showed that there was an increased callus formation in the ultrasound group. In cytological study, thymidine incorporation was found to be increased in the ultrasound group.

**Conclusions:**

The results of animal and clinical studies demonstrated an early and increased callus formation in the ultrasound group. Cytological studies revealed increased thymidine incorporation, suggesting increased osteoblastic activity.

## INTRODUCTION

Ultrasound has been used therapeutically for accelerating fracture healing for the past many years. Ever since its clinical use for the first time, there was a growing interest in this modality of osteoinduction. However, the controversy on the exact mechanism of osteoinduction still continues. The US Food and Drug Administration (FDA) has approved the use of ultrasound in fresh fractures (1994)^1^,^2^ and for established nonunions (2000).^3^,^4^ The role of chemical messengers like neurotransmitters in osteoinduction has evoked new research interest in this field.^5^–^7^ In this study, we try to bring out the exact biomolecular mechanism by which ultrasound induces fracture healing. If we could elucidate the exact mechanism of osteoinduction at the cellular or molecular level, we may be able to modify fracture healing as such.

## MATERIALS AND METHODS

**Figure 1:**
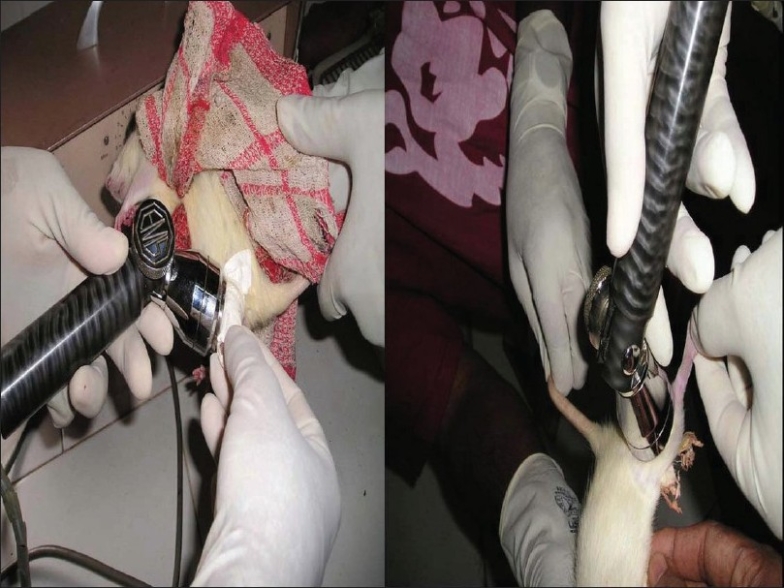
Therapeutic ultrasound in test group

**Figure 2:**
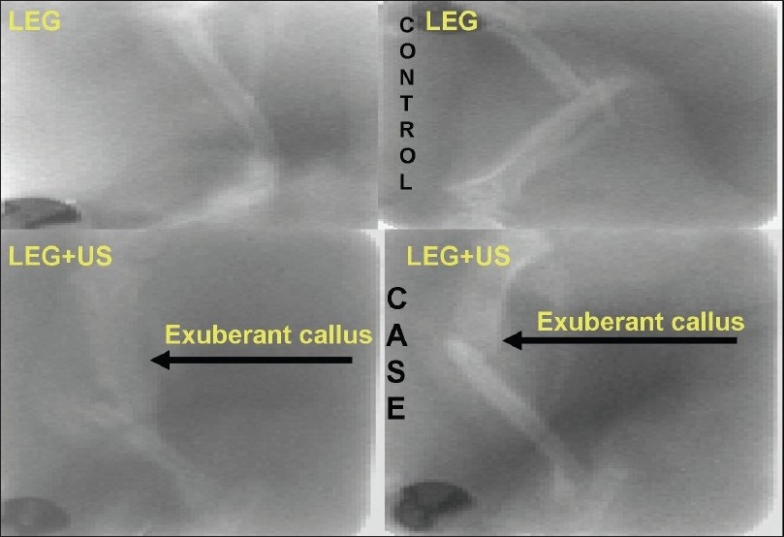
Radiological evaluation at 2 weeks. Note the exuberant callus in the test group

The study protocol was cleared by the institutional review board. The study was conducted in two phases. In the first phase, we induced fractures of the left tibia in Wistar strains of rats under ether anaesthesia. The fractures were splinted with special plastic splints. The rats were divided into two groups. The first group of 10 rats, defined as test group, was given low-intensity, pulsed ultrasound (30 MW/cm^2^) 20 min a day for 10 days. In the second group, of 10 rats, defined as control group, ultrasound treatment was not offered. Tissue samples and serial radiographs were obtained weekly for 3 weeks from both groups. The tissue samples were taken by aspiration from the fracture site [Figures 1 and 2].

Cells were taken in the phosphate buffer solution (PBS), and their viability was tested with Trifan blue stain. Then the cells were cultured in Rosewaal Pasteur Memorial Institute (RPMI) medium for 24 h. Radioactive thymidine^8^ incorporation test was done on these cells. The tissue aspirated from the fracture site was incubated with radioactive thymidine, and radioactivity was measured in a scintillation counter. The results of the uptake study in the test and control groups were compared statistically. Cells were again cultured with various neurotransmitter combinations, such as serotonin (5HT), dopamine (D)+, butaclomol (BU), GABA+ bicuculline (BI), and radioactive thymidine incorporation test was done; the results compared between both groups.

In the second phase of the study, 10 patients with fracture of the distal radius (extraarticular simple fractures) were included. Five of these fractures were designated as test group, and the rest five were treated as controls. The test group was subjected to low-intensity, pulsed ultrasound applied at the fracture site 20 min daily for 2 weeks. Those belonging to the control group were not given ultrasound therapy. The patients were assessed radiologically, sonologically, and through cell studies before and after ultrasound therapy exactly as in the first phase. The cytological study was conducted with the aspirate from the fracture site exactly as in the first phase.

## RESULTS

In the first phase of study, there was an increased callus formation in the ultrasound group when assessed radiologically by measuring the dimensions of the callus at the fracture site in the anteroposterior and lateral views using a scale.^9^–^11^ At the cellular level, there was an increased thymidine incorporation in the ultrasound group, as expressed by the scintillation counts shown in Tables 1–3.

**Table 1:** Radioactive thymidine incorporation test expressed in scintillation counts

	Test	Control
First week	8737	7190
Second week	8863	6583
Third week	9285	7814

**Table 2:** Radioactive thymidine incorporation test with neurotransmitters

Neurotansmitters	1 week		2 week		3 week	
			
	Test	Control	Test	Control	Test	Control
Serotonin	9499	119	6674	6607	8490	7752
Dopamine + Butaclomol	9510	8622	9613	8061	7582	6161
GABA + Bicuculline	10403	2451	8916	6447	9030	6228

**Table 3:** Radioactive thymidine incorporation test in cells after administration of 5HT, D+BU and GABA+BI

Patient	RTI in patients		RTI in cells after 5HT administration		RTI in cells after D + BU administration		RTI in cells after GABA + BI administration	
				
	Test	Control	Test	Control	Test	Control	Test	Control
1	8750	5679	11 006	7486	11 608	7184	9433	5936
2	9319	4357	79 970	6052	56 605	7200	70871	5285
3	7844	4532	32 533	6573	33 832	6943	33062	5487
4	6567	4578	43 453	5489	41 326	5467	45632	4378
5	7786	5371	39 763	6913	39 552	4774	37194	5279

RTI: Radioactive thymidine incorporation

**Figure 3:**
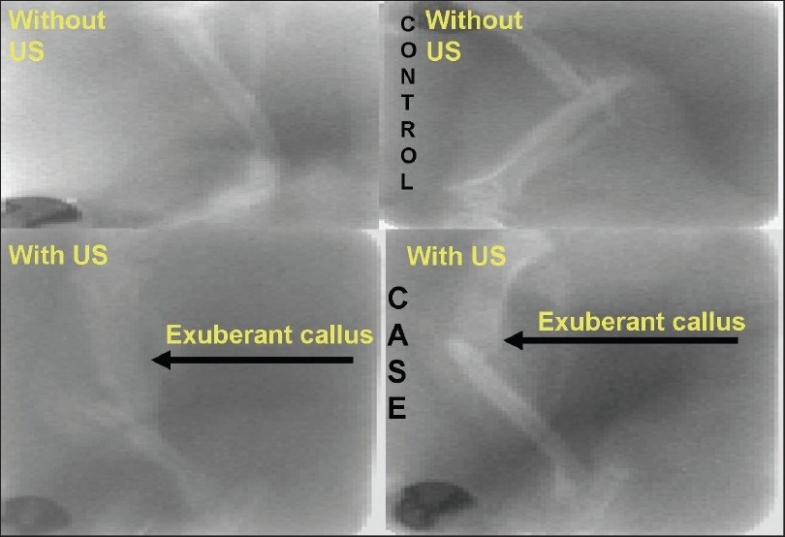
Difference in callus observed without ultrasound and with ultrasound

**Graph 1:**
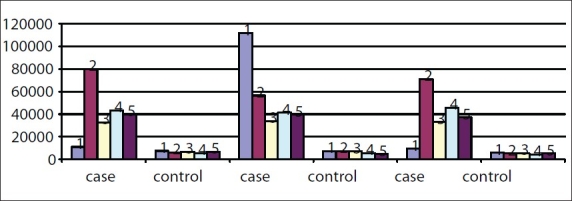
Radioactive study after neurotransmitter administration composite comparison. 5HT D + BU GABA + BI

In the second phase of the study, there was an increased callus formation when assessed sonologically in the ultrasound group [Figure 3]. And, at the cellular level, there was increased thymidine incorporation in the ultrasound group. By using the radioactive thymidine incorporation test [Graph 1], increased osteoblastic activity was observed when neurotransmitter combinations were added to the cells from fracture site in ultrasound group.

These experimental and clinical observations strongly suggest that the radioactive thymidine incorporation was significantly higher in the test group after ultrasound therapy and after neurotransmitter administration.

## DISCUSSION

Low-intensity, pulsed ultrasound has been proved in previous studies in the literature to significantly enhance fracture healing. In our study, the level of radioactivity^12^,^13^ was an index of osteoblastic activity at the fracture site. In the test group, the radioactive^12^ thymidine incorporation was significantly higher when compared with that in the control group. This is a definite evidence to suggest that ultrasound increases cell replication at the fracture site.

We tried to elucidate the mechanism by which ultrasound enhances healing by experimentally adding neurotransmitters at the fracture site in both the test group and controls. The radioactive thymidine incorporation was markedly increased in the test group with all the three different sets of neurotransmitters added.^11^ Because the neurotransmitters act through their respective receptors, this might indicate that the receptor levels of these neurotransmitters were significantly increased at the fracture site by ultrasound administration.^14^

Thus, we postulate that ultrasound enhances fracture healing by increasing the receptor activity of neurotransmitters and hence the neurotransmitter activity at the fracture site. This also indicates that neurotransmitters play a role in inducing fracture healing. How neurotransmitters act at the molecular or subcellular level in enhancing the healing process remains to be studied in detail. Also the present study has to be supplemented by further studies in larger populations to authenticate the proposed mechanism by which ultrasound induces fracture healing. In the light of the concrete base thus achieved, this could pave the way for an entirely new frontier in fracture management and impetus for further research.

## CONCLUSION

We conclude that low-intensity, pulsed ultrasound acts by upregulating the receptor activity of neurotransmitters at the fracture site, which probably enhances fracture healing.
